# Syphilis and Hepatitis B Co-infection among HIV-Infected, Sex-Trafficked Women and Girls, Nepal

**DOI:** 10.3201/eid1406.080090

**Published:** 2008-06

**Authors:** Jay G. Silverman, Michele R. Decker, Jhumka Gupta, Ashwin Dharmadhikari, George R. Seage, Anita Raj

**Affiliations:** * Harvard School of Public Health, Boston, MA, USA; † Brigham & Women's Hospital, Boston; ‡ Boston University School of Public Health, Boston

**Keywords:** HIV, STI, co-infection, India, Nepal, sex trafficking, dispatch

## Abstract

Sex trafficking may play a major role in spread of HIV across South Asia. We investigated co-infection with HIV and other sexually transmitted diseases among 246 sex-trafficked women and girls from Nepal. Those who were HIV positive were more likely than those who were HIV negative to be infected with syphilis and/or hepatitis B.

South Asia is currently home to >2.5 million HIV-infected persons, 95% of whom are from India (*1*). However, HIV seroprevalence in a subset of neighboring South Asian countries has rapidly increased in recent years, due in part to migration and human trafficking from these countries into India (*1*–*3*). Female sex workers, especially those who are victims of sex trafficking to India, are increasingly recognized as a major factor in Nepal’s growing HIV epidemic (*2*,*4*,*5*). HIV seroprevalence among female sex workers in Nepal rose 24-fold (from <1% to 17%) from 1992 through 2002 (*6*). Women and girls trafficked for sexual exploitation from Nepal to India are considered particularly vulnerable to HIV infection because of their typically young age at trafficking, limited ability to negotiate condom-protected sex, and experiences of forced sex (*4*,*7*). Recent evidence documents high (38%) HIV seroprevalence among sex-trafficked women and girls returning from India to Nepal (*5*).

Despite high rates of HIV infection among sex-trafficked victims (*5*,*8*) and substantial prevalence of sexually transmitted infections (STIs) among female sex workers in South Asia and elsewhere (*9*,*10*), little is known about STI prevalence and co-infection with HIV among sex-trafficked women and girls. We therefore explored prevalence of syphilis and hepatitis B and co-infection with HIV among a sample of female sex-trafficking victims in Nepal.

## The Study

Data for this study were extracted from medical records of 395 women and girls examined at Maiti Nepal, a large nongovernment organization in Kathmandu, Nepal, which provides shelter and healthcare for sex-trafficking victims repatriated to Nepal. Upon intake and pending verbal consent, all sex-trafficking victims at Maiti Nepal are routinely tested for HIV and STIs. We excluded records of 149 because they lacked HIV test or accompanying syphilis or hepatitis B test documentation. Study protocols were approved by the Harvard School of Public Health Human Subjects Committee.

Standard HIV antibody testing was performed by using HIV ELISA, rapid testing, or Western blot. Syphilis testing was performed by using a nontreponemal serologic test, the Venereal Disease Research Laboratory test; all samples tested had titers >1:8 dilution, which strongly suggests true syphilis infection (*11*). Serologic detection of the hepatitis B virus surface antigen was indicative of hepatitis B infection.

Age at time of HIV testing ranged from 13 to 40 years (median age 20 years), median age at the time of trafficking was 17 years (range 7–32 years), and median duration of brothel servitude was 12 months (range <1 month–13 years). A series of Fisher exact tests conducted to assess for potential biases in selection for diagnostic testing showed no differences in demographic or experiential variables (i.e., current age, age at trafficking, duration in brothel), and McNemar tests showed no relationship between likelihood of testing for syphilis or hepatitis B based on a positive HIV test result (all p>0.05). Because of the paired nature of the data, the McNemar test involving a continuity correction was used to assess associations between 1) HIV status and co-infection with syphilis, 2) HIV status and co-infection with hepatitis B, and 3) HIV status and co-infection with hepatitis B or syphilis.

Of the 246 women and girls in the study, 74 (30.1%, ≈1 in 3) had positive HIV test results. Syphilis infection was documented for 48 of 235 (20.4%, 1 in 5). Hepatitis B infection was documented for 8 of 210 (3.8%, 1 in 25). Those who were HIV positive were more likely than those who were HIV negative to be infected with syphilis (31.0% vs. 15.9%, respectively; odds ratio [OR] 1.88; 95% confidence interval [CI] 1.17–3.03) (Table 1). Similarly, those who were HIV positive were more likely than those who were HIV negative to be infected with hepatitis B (9.1% vs. 1.4%, respectively; OR 30.0; 95% CI 7.32–122.7) (Table 1). Finally, those who were HIV positive were more likely than those who were HIV negative to be infected with either syphilis or hepatitis B (35.1% vs. 15.1%, respectively; OR 1.78; 95% CI 1.11–2.85) (Table 2).

**Table 1:** Prevalence and likelihood of infection with syphilis or hepatitis B, by HIV serostatus, among sex-trafficked women and girls, Nepal

Syphilis (n = 235)	HIV status, no. (%)*	Syphilis positive, no. (%)†	Syphilis negative, no. (%)†	McNemar test statistic	OR (95% CI)†
HIV positive	71 (30.2)	22 (31.0)	49 (69.0)	6.45, p<0.05	1.88 (1.17–3.03)
HIV negative	164 (69.8)	26 (15.9)	138 (84.1)		Referent
Total	235	48 (20.4)§	187(79.6)§		
Hepatitis B (n = 210)		Hepatitis B positive, no. (%)†	Hepatitis B negative, no. (%)†		
HIV positive	66 (31.4)	6 (9.1)	60 (90.9)	52.4, p<0.0001	30.0 (7.32–122.73)
HIV negative	144 (68.6)	2 (1.4)	142 (98.6)		Referent
Total	210	8 (3.8)‡	202 (96.1)‡		

*Percentages based on HIV serostatus. †OR, odds ratio, CI, confidence interval. ‡Percentages based on total sample tested for HIV and syphilis or hepatitis B.

**Table 2:** Prevalence and likelihood of co-infection with syphilis or hepatitis B based on HIV serostatus (n = 246), among sex-trafficked women and girls, Nepal

HIV status	HIV status, no. (%)	Syphilis and/or hepatitis B positive, no. (%)*	Syphilis and hepatitis B negative, no. (%)*	McNemar test statistic	OR, 95% CI†
Positive	74 (30.1)	26 (35.1)	48 (64.9)	5.33, p<0.05	1.78 (1.11–2.85)
Negative	172 (69.9)	27 (15.7)	145 (84.3)		Referent
Total	246	53 (21.5)‡	193 (78.5)‡		

*Percentages based on HIV serostatus. †OR, odds ratio, CI, confidence interval. ‡Percentages based on total sample tested for HIV, syphilis, or hepatitis B.

## Conclusions

Our findings demonstrate that HIV-infected sex-trafficking victims are more likely to be infected with other STIs, specifically syphilis and hepatitis B, than those not infected with HIV. Current evidence of HIV and STI co-infection implies a need to strengthen clinical practice among providers caring for persons at risk for HIV or other STIs, particularly high-risk populations such as those trafficked for sexual exploitation or otherwise exposed to commercial sex work. Our findings strongly indicate the need for syphilis and hepatitis B screening for HIV-infected persons and HIV screening for syphilis- and hepatitis B-infected persons. Clinical expertise alone may be insufficient to guide treatment decisions in the presence of undetected co-infection (*12*,*13*), resulting in missed case detection, incomplete or partial treatment, and suboptimal clinical follow-up.

Appropriate diagnosis of co-infection by comprehensive STI and HIV screening is also important for averting potential development of pathogen drug resistance, a disastrous scenario in a region that is already coping with high rates of syphilis, hepatitis B, and HIV infection. From a clinical perspective, accurate diagnosis of syphilis, hepatitis B, and HIV, alone or in combination, is critical for informed selection of medications to be used in combinations or regimens that reduce the likelihood of inciting drug resistance for the other pathogens. Furthermore, success of STI treatment depends not only on the potency of the antiviral medication but also on the patient’s immunocompetence (*14*). A decision about when to modify a potentially failing or failed STI treatment regimen may thus be better informed by knowledge of HIV status.

Current data highlight prior calls for secondary prevention efforts (i.e., prevention of subsequent transmission) for this population because migration and repatriation of such women and girls has been described as a major factor in the spread of HIV and STIs across South Asia (*4*). Evidence from the World Health Organization shows that effective treatment of a variety of STIs can reduce HIV transmission rates because many STIs are increasing the risk for HIV acquisition (*15*). Therefore, treatment of prevailing STIs at the time of repatriation could potentially lessen risk for future HIV acquisition and reduce subsequent transmission to sex partners. The ability to properly treat and reduce the propagation of STIs represents an avenue by which to improve the health of the individual patients as well as potentially reduce rates of HIV in the region.

Finally, these data underscore the need for efforts by both government and nongovernment organizations to expand support for appropriate healthcare services to sex-trafficked women and girls and to develop comprehensive screening guidelines and treatment programs. Currently, most of the few nongovernment organizations serving this vulnerable population are unable to provide the quality of care indicated by our findings. Such improvements are urgently needed to help reduce the alarmingly high rates of HIV and co-occurring STIs among victims of sex trafficking and to curb the spread of these co-occurring epidemics throughout the region.

## References

[1] Joint United Nations Programme on HIV/AIDS/World Health Organization. Report on the global AIDS epidemic. Geneva: The Organization; 2006.

[2] Joint United Nations Programme on HIV/AIDS. Nepal country profile. Kathmandu (Nepal): The Programme; 2006.

[3] Joint United Nations Programme on HIV/AIDS. India country profile. Delhi (India): The Programme; 2006.

[4] Huda S. Sex trafficking in South Asia. Int J Gynaecol Obstet. 2006;94:374–81. 10.1016/j.ijgo.2006.04.027 16846602

[5] Silverman JG, Decker MR, Gupta J, Maheshwari A, Willis BM, Raj A. HIV prevalence and predictors of infection in sex-trafficked Nepalese girls and women. JAMA. 2007;298:536–42. 10.1001/jama.298.5.536 17666674

[6] Joint United Nations Programme on HIV/AIDS/World Health Organization. Epidemiological fact sheets on HIV/AIDS and sexually transmitted infections, 2004 update, Nepal. Geneva: The Organization; 2004.

[7] US Department of State. Victims of Trafficking and Violence Protection Act of 2000: trafficking in persons report. Washington: The Department; 2005.

[8] Silverman JG, Decker MR, Gupta J, Maheshwari A, Patel V, Raj A. HIV prevalence and predictors among rescued sex-trafficked women and girls in Mumbai, India. J Acquir Immune Defic Syndr. 2006;43:588–93. 17019369 10.1097/01.qai.0000243101.57523.7d

[9] Desai VK, Kosambiya JK, Thakor HG, Umrigar DD, Khandwala BR, Bhuyan KK. Prevalence of sexually transmitted infections and performance of STI syndromes against aetiological diagnosis, in female sex workers of red light area in Surat, India. Sex Transm Infect. 2003;79:111–5. 10.1136/sti.79.2.111 12690130 PMC1744644

[10] Sarkar K, Bal B, Mukherjee SK, Niyogi SK, Saha MK, Bhattacharya SK. Epidemiology of HIV Infection among brothel-based sex workers in Kolkata, India. J Health Popul Nutr. 2005;23:231–5. 16262019

[11] World Health Organization. Sexually transmitted and other reproductive tract infections: a guide to essential practice. Geneva: The Organization; 2005.

[12] Rompalo AM, Joesoef MR, O’Donnell JA, Augenbraun M, Brady W, Radolf JD, Clinical manifestations of early syphilis by HIV status and gender: results of the syphilis and HIV study. Sex Transm Dis. 2001;28:158–65. 10.1097/00007435-200103000-00007 11289198

[13] Rompalo AM, Lawlor J, Seaman P, Quinn TC, Zenilman JM, Hook EW III. Modification of syphilitic genital ulcer manifestations by coexistent HIV infection. Sex Transm Dis. 2001;28:448–54. 10.1097/00007435-200108000-00004 11473216

[14] Tillmann HL. Screening for and treating hepatitis B virus in patients with HIV infection. Clin Infect Dis. 2007;45:633–6. 10.1086/520753 17683000

[15] World Health Organization. Guidelines for surveillance of sexually transmitted diseases. New Delhi (India): The Organization; 2000.

